# Description of interference in the measurement of troponin T by a high-sensitivity method

**DOI:** 10.11613/BM.2019.021001

**Published:** 2019-06-15

**Authors:** Miguel Aliste-Fernández, Gemma Sole-Enrech, Ruth Cano-Corres, Silvia Teodoro-Marin, Eugenio Berlanga-Escalera

**Affiliations:** 1 Clinical Laboratory, Biochemistry Department, Parc Taulí Hospital Universitari, Institut d’Investigació i Innovació Parc Taulí I3PT, Universitat Autònoma de Barcelona, Sabadell, Spain; 2 Pediatric Medicine, Parc Taulí Hospital Universitari, Institut d’Investigació i Innovació Parc Taulí I3PT, Universitat Autònoma de Barcelona, Sabadell, Spain

**Keywords:** high-sensitivity troponin T, interference, case report

## Abstract

**Introduction:**

Measurement of high-sensitivity troponin T (hs-TnT) has become an essential step in the diagnosis of acute myocardial infarction. This high-sensitivity method allows quantifying the concentration of troponin T in blood of healthy subjects with a lower inaccuracy compared to previous reagent generations. However, the presence of certain compounds in the sample may interfere with the result. We present a patient who had repeatedly high concentrations of hs-TnT in the serum sample that did not agreed with the signs and symptoms. In addition, ultrasensitive troponin I concentration was undetectable.

**Materials and methods:**

To investigate the presence of an interfering compound, different analysis were carried out. In order to discard macro complexes in the sample, the serum was precipitated with polyethylene glycol. In addition, the serum was incubated with Scantibodies Heterophilic Blocking Tube, which can block heterophilic antibodies. Finally, a size exclusion chromatography of the sample was performed by the manufacturer.

**What happened:**

The interfering substance was allocated into fractions with proteins of 150kDa, corresponding to high molecular weight proteins like immunoglobulin G (IgG). This compound was responsible for the falsely elevated hs-TnT results and it affected only the high-sensitivity methods.

**Main lesson:**

The detected interfering compound was probably an IgG. This type of interference must be kept in mind in front of discordant results, even if they are extremely rare. Therefore, interdisciplinary cooperation between clinicians, laboratory and manufacturer is essential.

## Introduction

The use of the new high-sensitivity troponin (hs-Tn) tests has changed the approach to the diagnosis of the acute myocardial infarction (AMI). These methods of high sensitivity are characterized by the improvement of the detection limit, which is defined as the lowest concentration of analyte detectable by analytical method (*1*). As a result, it is possible to measure the concentration of troponin in blood of healthy subjects with higher sensitivity and lower inaccuracy compared to previous reagent generations, which in turn allows a better definition of the 99^th^ percentile. It is useful in particular to detect earlier troponin changes after the acute event. Since other cardiac and no cardiac pathologies can slightly increase the troponin concentration the recent measurement improvement reduces the specificity for the diagnosis of AMI (*2*). Moreover, interfering compounds can alter the measurement of troponin concentration in the sample, obtaining falsely increased or decreased results. Due to the complexity of the antigen-antibody interaction, immunoassays are particularly susceptible to these kinds of interferences, but optimizations in hs-Tn assays designs led to reported outlier rates of < 0.6% (*3*).

## Case description

A 12-years-old boy was admitted to the hospital with episodes of chest pain triggered in effort and repose. He was subjected to an electrocardiogram and echocardiogram with normal results. The laboratory tests showed an elevation of high-sensitivity troponin T (hs-TnT) of 51 ng/L (99th percentile = 14 ng/L), creatine kinase (CK) within the reference range and preserved renal function. Exercise test was within normality range and thus acute process by ischemia, pericarditis or myocarditis was discarded.

Blood tests were repeated 4 months later and an increase of hs-TnT up to 98 ng/L was found, while CK and other parameters were within normal ranges. A second echocardiography was performed and no pathological alterations were detected.

A new blood sample was collected one month later and high concentration of hs-TnT (52 ng/L) was observed again. Given the absence of compatible symptoms, a falsely positive result caused by interference was suspected.

## Laboratory analyses

All patient samples were drawn in gel separator tubes without anticoagulant (Vacuette Z serum sep clot activator, Greiner Bio-One, Kremsmünster, Austria). Tubes were left at room temperature for 15 minutes approximately to allow blood coagulation and then centrifuged at 2030xg for 10 minutes. Both analysers for measuring the concentration of hs-TnT available in our laboratory, Cobas e801 and Cobas e601 (Roche Diagnostics Mannheim, Germany), employ electrochemiluminescence immunoassays (ECLIA) and use a sandwich method with two mouse monoclonal antibodies (capture and detection), which are specifically directed against epitopes 125-131 and 136-147 of human cardiac troponin T (*4*).

Precicontrol Troponin quality control (ref: 05095107922, Roche Diagnostics, Mannheim, Germany) was processed during the study period for both analysers. The coefficient of variation (CV) obtained during this period was 3.0% for Cobas e601 and 3.5% for Cobas e801.

The first two serum samples from the patient were only processed on the Cobas e801 analyser, while the third one was processed on both analysers and sent to an external laboratory to measure the concentration of high-sensitivity troponin I (hs-TnI) on the Architect-i System platform (Abbott Diagnostics, Chicago, USA), using the hs-TnI STAT reagent. However, it is important to take into account that the interference susceptibility might vary among the different measurement systems (*e.g.* even among various hs-TnI assays) (*5*).

Referring to the literature on the study of analytical interferences, we followed the recommendations described by Ward *et al.* and other publications with similar cases, to identify potential interfering factors such as fibrin micro clots, haemolysis or human anti-mouse antibodies (*3*, *6*). Firstly, we ruled out pre-analytical problems and we performed a serial dilution of the sample using the “Diluent MultiAssay” (Roche Diagnostics GmbH, Mannheim, Germany). The concentration of hs-TnT was measured on the Cobas e801 automated platform. We also performed a polyethyleneglycol (PEG) precipitation (Merck-Schuchardt, Hohenbrunn, Germany) of the serum to rule out the presence of macrocomplexes, by mixing 300 µL of the serum sample with the same volume of a solution of PEG 6000 at 25%. Then, the mixture was centrifuged at 1500xg for 20 minutes and supernatant was assayed.

Since mouse antibodies are also used in the measurement procedure, other analytes which could potentially be influenced by the same interference were measured. In addition, the concentrations of immunoglobulins and rheumatoid factor were measured employing an automated nephelometric assay (Immage 800, Beckman Coulter, Brea, USA).

In order to confirm the results, another visit was scheduled 3 months later in which all tests were repeated and the concentration of various autoantibodies by indirect immunofluorescence assay (Quantalyser 160, Werfen, California, USA) was measured (Table 1). In addition, the presence of heterophilic antibodies in the sample was assessed by incubation for 1 hour with the Scantibodies Heterophilic Blocking Tube (HBT) (Scantibodies Laboratory, Santee, USA), which can block these antibodies.

**Table 1:** Measurements performed to elucidate the cause of the falsely elevated results of hs-TnT

**Possible causes**	**Our results**	**Reference ranges**	**Analyser**
**Interference by endogenous compounds in blood (haemoglobin, bilirubin and lipemia)**	Serum indices: Haemolysis (H) = 8 Icterus (I) = 1 Lipemia (L) = 4	No significant interference up to: H = 100 mg/dL I = 25 mg/dL L = 1500 mg/dL	Cobas 8000
**Cross reactivity with skeletal muscle**	Creatine kinase: 152 U/L	0-195 U/L	Cobas 8000
**High activity of alkaline phosphatase**	Alkaline phosphatase: 150 U/L	2-300 U/L	Cobas 8000
**Kidney failure**	Serum creatinine: 52 µmol/L	34–65 µmol/L	Cobas 8000
**Macro complex in the sample (Concentration of hs-TnT)**	Sample before reaction: 52 ng/L Sample after reaction with PEG 6000: 67 ng/L Percent recovery > 100%	-	Cobas e801
**Measurements of other analytes which could potentially influence the same interference**	PTH: 14.4 ng/L CA 125: 19.8 kU/L AFP: under detection limit CA 19.9: 12.6 kU/L TPSA: under detection limit TSH: 4.44 mIU/L FT4: 19.8 pmol/L	10.0-65.0 ng/L < 35.0 kU/L < 6.0 kUI/L < 22.0 kU/L < 4.0 µg/L 0.40-5.00 mIU/L 10.3–23.2 pmol/L	Cobas e801
**Concentration of immunoglobulins**	Immunoglobulin G: 14.30g/L Immunoglobulin M: 1.52 g/L Immunoglobulin A: 1.35 g/L	7.40–14.00 g/L 0.70-2.00 g/L 0.60-3.56 g/L	Immage 800
**Rheumatoid factor**	< 20 IU/mL	70–200 IU/mL	Immage 800
**Autoantibodies titers**	ANA, ASMA, AMA and APCA: < 1/40 ANCA: Negative	< 1/40 Negative	Quantalyser 160
**Heterophile antibodies (Concentration of hs-TnT)**	Sample before incubation: 32 ng/L Sample after incubation with HBT: 33 ng/L Absence of heterophile antibodies removable by HBT	-	Cobas e601
hs-TnT - high-sensitivity troponin T. HBT - heterophilic blocking tube. PTH - parathyroid hormone. CA - carbohydrate antigen. AFP - alpha-fetoprotein. TPSA - total specific prostate antigen. TSH – thyrotropin. FT4 - free thyroxine. ANA - anti-nuclear antibodies. ASMA - anti-smooth muscle antibodies. AMA - anti-mitochondrial antibodies. APCA - anti-parietal cells antibodies. ANCA - anti-neutrophil cytoplasmic antibodies.			

Finally, the serum sample was sent to the manufacturer to further investigate the potential interference. The sample was fractionated by size exclusion chromatography to detect the molecular size that could cause interference with the hs-TnT test. The principle of this technique includes a different elution speed depending on the molecular weight. Therefore, since the molecular weight of troponin T is known (~36 kDa), it is possible to infer if the fraction in which troponin T is expected is also showing the reactivity with the hs-TnT assay.

## What happened?

Table 2 shows the evolution of the troponin T concentration in the patient, as well as other biomarkers of myocardial injury.

**Table 2:** Evolution of the different myocardial damage markers in the patient; single reading

**Visit**	**Analyte (unit); analyser**			
	**CK (U/L);** **Cobas 8000**	**hs-TnT (ng/L);** **Cobas e801**	**hs-TnT STAT (ng/L);** **Cobas e601**	**hs-TnI STAT (ng/L); Architect**
**First visit**	115	51	-	-
**Second visit**	49	98	-	-
**Third visit**	152	52	28	< 10
**Fourth visit**	129	-	32	< 10
CK - creatine kinase. hs-TnT - high-sensitivity troponin T. STAT - short turnaround time. hs-TnI - high-sensitivity troponin I. The 99th percentile for the specific test was: 0 - 195 U/L (CK), ≤ 14 ng/L (hs-TnT and hs-TnT STAT), ≤ 26 ng/L (hs-TnI STAT).				

**Figure 1:**
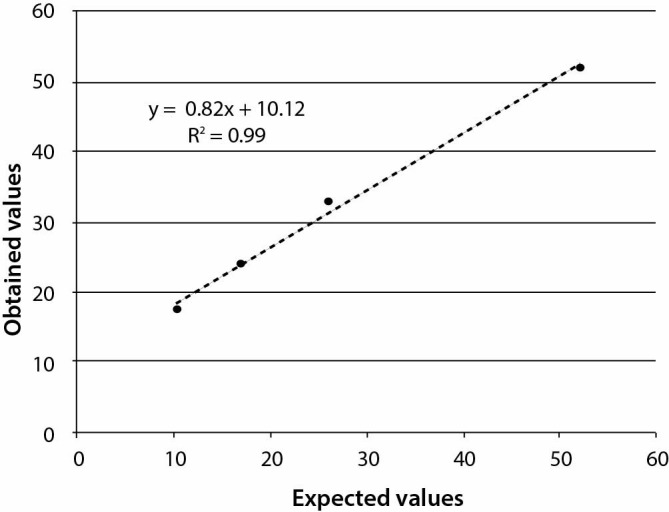
Linearity test between expected and obtained values of the dilution test (ng/L).

According to the literature on interference in immunoassays additional studies were performed, as summarized in Table 1 (*3*, *6*). Regarding the dilution test, expected and observed values are shown in Table 3. Moreover, a linearity test between expected and observed values was performed (Figure 1), which showed that existed linearity between them (R^2^ = 0.99).

**Table 3:** Results of dilution test (Cobas e801)

	**Sample before dilution**	**Dilution 1:2**	**Dilution 1:3**	**Dilution 1:5**
**Concentration of high-sensitivity troponin T measured** **(Dilution result x DF)**	52 ng/L	33 ng/L (66 ng/L)	24 ng/L (72 ng/L)	18 ng/L (88 ng/L)
**Concentration of high-sensitivity troponin T expected**	52 ng/L	< 26 ng/L	< 17 ng/L	< 10 ng/L
DF – dilution factor.				

Size exclusion chromatography results suggest that the interference can be allocated to fractions with proteins of 150 kDa, corresponding to high molecular weight proteins like immunoglobulin G (IgG). Quoting the manufacturer report: “the observed hs-TnT values are falsely elevated in the sample. The interference is caused by a high molecular weight compound, presumably IgG”. Independent studies concluded that such type of interference are rare and are covered by disclaimers in the method sheet (*3*). The manufacturer also performed troponin T measurements in different analysers (N = 1) employing ECLIA technology (Table 4).

**Table 4:** Manufacturer’s study in which troponin T concentration were measured with different analysers with electrochemiluminescence immunoassay technology

	**hs-TnT STAT**			**TnT STAT**	**hs-TnT**
**Analyser**	**e601 (ng/L)**	**e411 (ng/L)**	**e801 (ng/L)**	**e601 (ng/L)**	**e801 (ng/L)**
**Average of outcomes**	34	41	32	< 10	54
hs-TnT STAT - high-sensitivity troponin T short turnaround time. TnT STAT - troponin T short turnaround time. hs-TnT - high-sensitivity troponin T.					

## Discussion

We report the case of a 12-years-old boy with high concentration of hs-TnT without evidence of myocardial injury. As a consequence of this discordance, a falsely positive result was suspected.

After checking the correct calibration status of the analyser and repeating the hs-TnT measurements, possible pre-analytical causes that could alter the results were studied. The presence of fibrin clots or micro particles was ruled out after inspection of the sample and besides that, the haemolysis, icterus and lipemia indices were found to be under the interference level (*7*). Finally, renal failure, which may increase troponin concentration, was discarded for the patient (*8*).

A simple way to detect interference is performing serial dilutions of the sample with a suitable solvent. Normally, in the event that interference is present, reported concentrations will not dilute linearly until the substance is eliminated (*9*). According to the results of the dilution study, slightly higher troponin concentrations were observed when dilutions were made, which could be caused by the imprecision added to the measurement due to the dilution factor. Since the concentration of hs-TnT did not decrease as expected and the linearity test between expected and obtained values showed that existed linearity, we discarded the presence of an interferent disappearing with the dilution.

After performing this dilution test, we could not detect the interference, although its presence was evident taking into account the observed difference between the concentration of hs-TnT (52 ng/L) and hs-TnI (< 10 ng/L). Troponin T and Troponin I are both elevated in case of myocardial injury, so an undetectable concentration of hs-TnI meant that there was no real injury and the elevation of hs-TnT was caused by an interfering compound.

In addition, the presence of macro complexes was investigated but after not observing different results in the sample before and after PEG 6000 addition, such type of interferent was excluded (*10*, *11*).

After discarding the presence of rheumatoid factor, an IgM autoantibody with IgG-binding capacity, we investigated another well-known cause of interference in immunoassays: heterophilic antibodies (HA). Its incidence in the population lies between 0.2 - 40% and it includes two specific circumstances: the so-called “true” HA, which are multispecific and low affinity antibodies, and human anti-animal antibodies (HAAA), which are high affinity antibodies against specific animal immunoglobulins (HAMA) (*7*, *12*). They are present in the blood after treatment with monoclonal antibodies or by contact with animals, among other causes (*8*, *13*). In the case of falsely positive results, the HA, in the sample, bridge capture and detection antibodies to mimic analyte binding. The activity of this type of antibodies cannot be completely ignored even if manufacturers add certain compounds in their reagents to reduce its influence (*4*).

It is possible to eliminate the HA by the incubation of the sample with HBT. However, since some HBT tubes are not reliable when immunoassays employs materials derived from mice, HA elimination is not always possible (*6*). In our case, no differences were observed in the concentration of hs-TnT after the HBT sample incubation.

According to the manufacturer’s additional investigation, the interference might be eventually due to an IgG, although a final IgG validation was not proven. This IgG could correspond to an antibody that was not removable with a HBT, and thus possibly a HAMA, that might be found in serum of animal workers or in patients receiving mouse monoclonal antibodies for therapy or imaging. In support of that hypothesis, the medical interview revealed that the patient had several pets a few years ago, including hamsters. Moreover, the concentration of IgG in the patient serum sample was elevated (Table 1).

However, it was not possible to identify the cause of the discrepancy between the results obtained from the analysers of our laboratory. The only methodological difference lies in the use of technology STAT (Short turnaround time) in the measurement procedure of Cobas e601, which reduces the analysis time from 18 minutes to 9, by reducing the number of incubations from two to one. It might be possible that an extended incubation time could favour the union of the IgG to the antibodies, and therefore enhancing the interference, justifying the higher concentration of hs-TnT observed in the method that uses two incubations instead of one, but further validation was not possible. Nevertheless, such single results should not be over interpreted.

In addition, a former less sensitive method (Troponin T STAT) provided no evidence of troponin elevation in the same patient sample (< 10 ng/L), which indicates that the high-sensitivity methods might be more susceptible to such type of interference. Irrespectively of the used immunoassay type, either one or two step assays, the frequency of such HAMA interference has been reported between 0.1 - 3.1% and may involve both Troponin I and Troponin T tests (*12*). Given that the hs-Tn assays have consistently shown a higher negative predictive value compared to the less sensitive tests the average clinical performance indicators of the troponin tests need to be taken into account for a conclusive benefit-risk analysis (*14*).

## Main lesson

Nowadays, troponin measurements are indispensable part of AMI definition and related guidelines for their interpretation are urgently required in daily clinical practice. When a high concentration of troponin is not compatible with the clinic signs and symptoms of the patient, the presence of an interfering factor in the sample should always be suspected.

In the case reported here, falsely elevated results of hs-TnT in two different analysers might have been caused by an IgG antibody that could not be removed after incubation with HBT. At low analyte concentration, this effect was observed only for the high-sensitivity methods of troponin T. In addition, the concentration of hs-TnT was lower when STAT technology was used, which could be explained by the lower incubation time. Even if this type of interference is disclaimed by the manufacturer, the present case described the track to substantiate such hypothesis.

Interference by HA is a problem that may affect the immunoassays, especially after the monoclonal antibody-based therapy use in recent years (*15*, *16*). Therefore, interdisciplinary cooperation between clinicians, laboratory and manufacturer is essential for the appropriate interpretation of the results.
